# Genetic and phenotypic diversity in *Burkholderia*: contributions by prophage and phage-like elements

**DOI:** 10.1186/1471-2180-10-202

**Published:** 2010-07-28

**Authors:** Catherine M Ronning, Liliana Losada, Lauren Brinkac, Jason Inman, Ricky L Ulrich, Mark Schell, William C Nierman, David DeShazer

**Affiliations:** 1 J. Craig Venter Institute, 9704 Medical Center Drive, Rockville, MD 20850, USA; 2 U.S. Army Medical Research Institute of Infectious Diseases, 1425 Porter Street, Fort Detrick, MD 21702, USA; 3 Department of Microbiology, University of Georgia, Athens, GA 30602, USA; 4 U.S. Department of Energy, Office of Biological and Environmental Research, SC-23.2/Germantown Building, 1000 Independence Avenue SW, Washington DC 20585-1290, USA

## Abstract

**Background:**

*Burkholderia *species exhibit enormous phenotypic diversity, ranging from the nonpathogenic, soil- and water-inhabiting *Burkholderia thailandensis *to the virulent, host-adapted mammalian pathogen *B. mallei*. Genomic diversity is evident within *Burkholderia *species as well. Individual isolates of *Burkholderia pseudomallei *and *B. thailandensis*, for example, carry a variety of strain-specific genomic islands (GIs), including putative pathogenicity and metabolic islands, prophage-like islands, and prophages. These GIs may provide some strains with a competitive advantage in the environment and/or in the host relative to other strains.

**Results:**

Here we present the results of analysis of 37 prophages, putative prophages, and prophage-like elements from six different *Burkholderia *species. Five of these were spontaneously induced to form bacteriophage particles from *B. pseudomallei *and *B. thailandensis *strains and were isolated and fully sequenced; 24 were computationally predicted in sequenced *Burkholderia *genomes; and eight are previously characterized prophages or prophage-like elements. The results reveal numerous differences in both genome structure and gene content among elements derived from different species as well as from strains within species, due in part to the incorporation of additional DNA, or 'morons' into the prophage genomes. Implications for pathogenicity are also discussed. Lastly, RNAseq analysis of gene expression showed that many of the genes in ϕ1026b that appear to contribute to phage and lysogen fitness were expressed independently of the phage structural and replication genes.

**Conclusions:**

This study provides the first estimate of the relative contribution of prophages to the vast phenotypic diversity found among the *Burkholderiae*.

## Background

*Burkholderia pseudomallei*, causal agent of the potentially fatal disease melioidosis, is a metabolically versatile soil organism that has been classified as a Category B biological threat by the CDC [1,2]. Relatively little is known about its pathogenesis, virulence factors, the extent of diversity in natural populations, and host response. *B. pseudomallei *genome plasticity has been associated with genomic island variation. The genome of *B. pseudomallei *K96243 (7.3 Mb), for example, features 16 genomic islands, at least three of which appear to be prophages [3]. It is unclear whether all of these putative prophages are active, although one (ΦK96243) was shown to be a productive bacteriophage. *B. pseudomallei *isolates are genetically quite diverse [4,5], and this heterogeneity may be due at least in part to the highly variable distribution of bacteriophages among strains [6]. Such differences may provide certain strains survival advantages in the environment and the host, as well as explain the variable clinical presentation of melioidosis.

Also raising concern as a potential biological weapon is the very closely related *B. mallei*, causal agent of the primarily equine disease known as glanders [7]. In contrast to *B. pseudomallei*, *B. mallei *is a highly specialized pathogen, not found outside of a mammalian host in nature. *B. mallei *is a host-adapted clone of *B. pseudomallei*, and all of the *B. mallei *genome is nearly identical to a set of genes within *B. pseudomallei *core genome. However, in addition to its core genome *B. pseudomallei *contains numerous contiguous gene clusters that were deleted from *B. mallei *during its evolution [8,9].

*B. thailandensis *is another closely related organism often found in the same environmental samples (soil and water of endemic melioidosis regions) as *B. pseudomallei *[10]. Unlike *B. pseudomallei *and *B. mallei*, *B. thailandensis *has very low virulence in most animal hosts, including humans. The ability to metabolize arabinose, and the corresponding loss of the arabinose assimilation operon from *B. pseudomallei*, phenotypically distinguishes *B. thailandensis *from *B. pseudomallei *[11]. The genes encoding arabinose assimilation may be considered as antivirulent, and their absence from *B. pseudomallei *(and *B. mallei*) may have allowed the development of the latter as pathogens [12]. *Burkholderia multivorans*, a member of the *Burkholderia cepacia *complex, is an opportunistic pathogen associated with infection in cystic fibrosis patients that is also found in soil environments [13].

The presence of prophages among bacterial isolates and their possible contribution to bacterial diversity is widespread. By carrying various elements contributing to virulence, prophages can contribute to the genetic individuality of a bacterial strain. This phenomenon has been reported in *Salmonella *spp [14] and *Lactobacillus *spp [15,16], among others. Prophage-associated chromosomal rearrangements and deletions have been found to be largely responsible for strain-specific differences in *Streptococcus pyogenes *[17] and *Xylella fastidiosa *[18]. Thus, temperate phages carrying foreign DNA can play a role in the emergence of pathogenic variants. Lateral gene transfer between phage and host genomes, and phage lysogenic conversion genes, can alter host phenotype through production of phage-encoded toxins and disease-modifying factors that affect virulence of the bacterial strain. Examples of such phage-encoded virulence factors abound in the literature, and include proteins associated with toxicity, antigenicity, invasion, intracellular survival, serum resistance, and adhesion [19]. Many of these factors are encoded by morons that are present variably across phage genomes and are thought to be regulated independently of the phage genes [20].

To estimate the contribution of prophages to genetic and phenotypic diversity of the species, we have isolated and sequenced five temperate bacgteriophages from *Burkholderia*, three from *B. pseudomallei *and two from *B. thailandensis*, and used bioinformatics techniques to search for putative prophage regions in the genomes of nine sequenced *B. pseudomallei *strains, six *B. mallei *strains, one *B. thailandensis *strain, three *B. multivorans *strains, and one *Burkholderia xenovorans *strain. While no prophages were detected in any of the *B. mallei *strains, a total of 24 putative prophages or prophage-like islands (PI) were identified in the other species. Sequences from the isolated phages and inferred prophages were compared with each other and with the 8 published phage sequences from *B. pseudomallei*, *B. thailandensis*, *B. cenocepacia*, and *B. cepacia*. As seen in other genera, the prophages among the *Burkholderiae *contribute to the genomic variability of the species and carry genes that could provide advantages in the environment and host adaptation.

## Methods

### Spontaneous bacteriophage production by lysogenic B. pseudomallei and B. thailandensis strains, host range studies, and UV induction experiments

Five bacteriophages were isolated and fully sequenced (Table 1A).

**Table 1:** Sources and descriptions of bacteriophage and putative prophage islands (PI) used in this study.

*A. Isolated bacteriophages*								
Phage (Acc #)	Source	Description	Size (Mb)	# ORFs	Head diameter (nm)	Tail (length × diameter) (nm)	Plaque diameter (mm)	pfu/mL
φ52237 (NC_007145)	Bp Pasteur 52237		37.6	47	55	155 × 23	1.5 - 2.0	3 × 10^6^
φ644-2 (NC_009235)	Bp 644	Australia; disease (ulcer)	48.7	71	60	190 × 9	1.0	3 × 10^3^
φE12-2 (NC_009236)	Bp E12-2	NE Thailand; soil	36.7	50	62	152 × 21	1.5 - 2.0	1 × 10^1^
φE202 (NC_009234)	Bt E202	NE Thailand; soil	35.7	48	65	140 × 21	1.5 - 2.0	2 × 10^5^
φE255 (NC_009237)	Bt E255	central Thailand; soil	37.4	55	64	143 × 21	0.5	2 × 10^3^

***B. Inferred prophages ***								
**Prophage-like island**	**Source**	**ORFs**	**Size (Mb)**	**# ORFs**	**Chromosome**	**Description**		

PI S13-1	Bp S13	BURPSS13_G0002-G0044; BURPSS13_I0965-I0971	38.0	48	I	putative prophage		
PI S13-2	Bp S13	BURPSS13_T0353-T0354; BURPSS13_K0001-K0007	9.5	9	II	prophage-like		
PI S13-3	Bp S13	BURPSS13_T0561-T0598	23.4	38	II	prophage-like		
PI Pasteur-2	Bp Pasteur 6068	BURPSPAST_Y0106-Y0135	42.4	30	I	putative prophage		
PI Pasteur-3	Bp Pasteur 6068	BURPSPAST_P0245-P0287	60.1	45	I	prophage-like		
PI 1655-1	Bp 1655	BURPS1655_F0102-F0150	36.9	48	I	putative prophage		
PI 406E-1	Bp 406E	BURPS406E_K0245-K0264	17.9	20	I	putative prophage		
PI 406E-2	Bp 406E	BURPS406E_R0182-R0256	62.9	73	I	putative prophage		
PI 1710b-1	Bp 1710b	BURPS1710B_1505-1536	47.0	32	I	putative prophage		
PI 1710b-2	Bp 1710b	BURPS1710B_1538-1604	61.1	67	I	putative prophage		
PI 1710b-3	Bp 1710b	BURPS1710B_3650-3669	63.0	45	I	prophage-like		
PI 688-1	Bp 668	BURPS668_A2331-A2390	41.1	60	I	prophage-like		
PI E264-1 (GI1)	Bt E264	BTH_I0091-I0119	49.1	26	I	putative prophage		
PI E264-2 (GI13)	Bt E264	BTH_II1325-II1368	33.1	41	II	prophage-like		
PI E264-3 (GI12)	Bt E264	BTH_II1011-II1070	52.0	62	II	putative prophage		
PI LB400-1	Bx LB400	Bxe_A3036-A3110	53.4	40	I	putative prophage		
PI CGD1-1	Bmul CGD1	BURMUCGD1_3398-3447	37.7	51	I	putative prophage		
PI CGD1-2	Bmul CGD1	BURMUCGD1_2149-2203	45.6	56	I	prophage-like		
PI CGD2-1	Bmul CGD2	BURMUCGD2_1176-1227	36.6	52	I	putative prophage		
PI CGD2-2	Bmul CGD2	BURMUCGD2_2461-2520	44.6	60	I	prophage-like		
PI CGD2-3	Bmul CGD2	BURMUCGD2_4590-4656	49.4	67	II	prophage-like		
PI 17616-1	Bmul ATCC 17616	Bmul_1771-Bmul_1998	236.3	217	I	putative prophage		
PI 17616-3	Bmul ATCC 17616	Bmul_3828-Bmul_3914	73.0	80	II	prophage-like		
PI 17616-4	Bmul ATCC 17616	Bmul_4831-Bmul_4876	39.4	44	II	prophage-like		
GI3 (N/A)	Bp K96243	putative prophage [3]	51.2	31	I	putative prophage		
GI15 (N/A)	Bp K96243	putative prophage[3]	35.1	38	II	putative prophage		

***C. Published bacteriophages***								
**Phage (Acc #)**	**Source**	**Description**	**Size (Mb)**	**# ORFs**	**Chromosome**	**Description**		

Φ1026b (AY453853)	Bp 1026b	Siphoviridae [6]	54.9	83	I (?)	prophage		
GI2; ΦK96243 (N/A)	Bp K96243	Myoviridae [3]	36.4	45	I	prophage		
ΦE125 (AF447491)	Bt E125	Siphoviridae [52]	53.4	71	I (?)	prophage		
BcepMu (AY539836)	*B. cenocepacia *J2315	Myoviridae (Mu-like) [30]	36.7	53	III	prophage		
Bcep22 (AY349011)	*B. cepacia*	Podoviridae	63.9	81	N/A	prophage		
Bcep781 (AF543311)	*B. cepacia*	Myoviridae; [30]	48.2	66	N/A	prophage		

Bacteriophage production and plaque formation by *B. pseudomallei *and *B. thailandensis *strains were assessed using *B. mallei *ATCC 23344 as an indicator strain, as described previously [6,21].

*B. pseudomallei *strains Pasteur 52237, E12, and 644 and *B. thailandensis *strains E202 and E255 were grown in LB broth for 18 h at 37°C with shaking (250 rpm). Overnight cultures were briefly centrifuged to pellet the cells, and the supernatants were filter-sterilized (0.45 mm). The samples were serially diluted in suspension medium (SM) [22], and the number of plaque forming units (pfu) was assessed using *B. mallei *ATCC 23344 as the host strain. Briefly, one hundred microliters of filter-sterilized culture supernatant was added to a saturated *B. mallei *ATCC 23344 culture, incubated at 25°C for 20 min, and 4.8 ml of molten LB top agar (0.7%) containing 4% glycerol was added. The mixture was immediately poured onto a LB plate containing 4% glycerol and incubated overnight at 25°C or 37°C. For ϕE202 host range studies, this procedure was followed using the bacteria listed in Additional file 1, Table S1. Bacteria were considered to be sensitive to ϕE202 if they formed plaques under these conditions and resistant if they did not. No bacterial species tested formed plaques in the absence of ϕE202.

For ϕE202 UV induction studies, one hundred microliters of saturated *B. thailandensis *E202 culture was used to inoculate two LB broth (3 ml) subcultures. One set of subcultures was incubated for 5 h without interruption. The other set of subcultures was incubated for 3 h, poured into sterile petri dishes in a class II biological safety cabinet, subjected to a hand-held UV light source (254 nm) for 20 sec (25 cm above the sample), pipetted back into culture tubes, and incubated for an additional 2 h. The titer of the filter-sterilized supernatants were determined by performing quantitative plaque assays on serially diluted samples.

### Negative staining

To determine morphotypes, bacteriophages were prepared from 20 ml of a plate culture lysate, incubated at 37°C for 15 min with Nuclease Mixture (Promega), precipitated with Phage Precipitant (Promega), and resuspended in 1 ml of Phage Buffer (Promega). The bacteriophage solution (~100 μl) was added to a strip of parafilm M (Sigma), and a formvar-coated nickel grid (400 mesh) was floated on the bacteriophage solution for 30 min at 25°C. Excess fluid was removed, and the grid was placed on a drop of 1% phosphotungstic acid, pH 6.6 (PTA) for 2 min at 25°C. Excess fluid was removed, and the specimen was examined on a Philips CM100 transmission electron microscope. Nickel grids were glow discharged on the day of use.

### Bacteriophage sequencing and annotation

Libraries were constructed from the genomic DNA from the bacteriophage isolates. Since the phage genomes were estimated to be 50 kb in size, sequencing, closure, and annotation was performed similar to that of a BAC sequence [23]. Each of the five isolated bacteriophages were completely sequenced to 10× coverage, closed, and annotated, and the sequences deposited in GenBank (Table 1A).

### Identification of putative prophages and prophage-like elements within strains

Presence of prophage sequence within sequenced genomes of nine *B. pseudomallei *strains, six *B. mallei *strains [8], three *B. multivorans *strains, *B. thailandensis *E264 [24], and *B. xenovorans *LB400 [25] (Additional file 1, Table S2) was inferred using a number of similarity measures previously described [26,27]. First, the protein set of each genome was searched against a non-redundant database of viral proteins using BLASTP [28] with a cutoff of e^-10^. Secondly, the annotation of each strain was searched for several virus-related keywords such as integrase, tail, capsid, portal, terminase, etc. Clustering of such proteins with proteins containing similarity to known phage proteins as identified by BLASTP, as well as the orientation of proteins within clusters was considered strong evidence for prophage presence. Finally, tRNA genes and attachment sites were examined. A tRNA sequence immediately flanking an integrase was considered to be a potential *att *site, particularly if an exact repeat of least a 10 bp of the tRNA was present within several thousand bp. Regions containing all of these factors (i.e., viral-like proteins clustered and in a specific orientation, and flanked by a tRNA/integrase on one end and an exact repeat of at least 10 bp of the tRNA on the other end) were considered putative prophage. Regions containing many of these characteristics but lacking one or more, usually an integrase or repeat sequence, were considered prophage-like. Some *att *sites are less than 10 bp and are difficult to spot so it is possible that some of the prophage-like elements may actually be true prophages. Prophage and prophage-like regions so inferred have been designated "PI-*strain*-1", "PI-*strain*-2", etc. (PI for Prophage Island), and are listed in Table 1B. Four of the *B. pseudomallei *strains represent two paired isolates from two separate patients, one strain isolated from an initial infection and the paired isolate from a re-emergent infection in the same patient.

Three of the 16 genomic islands previously identified in *B. pseudomallei *K96243 were included in the analysis, including ϕK96243 (GI2) and the putative prophages GI3 and GI15 [3]. Three prophage-like islands identified in *B. thailandensis *E264, GI1, GI12, and GI13, were also included in the analysis (Table 1B) [24]. Additionally, the published genome sequences of ϕ1026b from *B. pseudomallei *1026b [6], ϕE125 from *B. thailandensis *E125 [21], BcepMu from *B. cenocepacia *J2315 [29], Bcep22 from *B. cepacia*, and Bcep781 from *B. cepacia *[30], all of which are classified as dsDNA phages, were included for comparison (Table 1C).

### Comparative genome sequence analysis and phylogenetic tree construction

The program Dotter [31] was used to align nucleotide sequences of all isolated and putative prophage and prophage-like sequences and to identify initial groupings. To refine clusters, distance measures were calculated between all pairs of each of the 30 prophage and PI sequences. Reciprocal BLASTP comparisons of the translated protein sets were performed for each prophage/PI against all others. BLASTP distances between each pair were calculated according to the formula: 1-(number of significant hits between both genomes/total number of genes in both genomes) [32]. Distances were calculated using E value cutoffs of 1 × E^-01^, 1 × E^-05^, and 1 × E^-10^. FITCH with the global and jumble options was used to generate a phylogenetic tree from each of the three distance matrices derived from the BLASTP distances [33].

Calculation of local collinear blocks (LCB or synteny blocks) was done using progressive Mauve alignment [34] with default settings. Initial identification of morons was conducted in the Mauve alignments by searching for ORFs that disrupted the collinearity in LCBs. Confirmation of morons was done by (i) comparing % GC content of each ORF against the mean % GC of phage-specific genes (i.e., involved in structure, replication, and host lysis); (ii) promoter and terminator prediction analysis with BPROM http://www.Softberry.com, PPP http://bioinformatics.biol.rug.nl/websoftware, PROMSCAN [35] or Promoter Prediction by Neural Network [36]; (iii) prediction of terminators with TransTermHP [37]; and (iv) search for homologs across different phage types within our data set and in the non-redundant database at NCBI.

### Phage gene expression analysis using RNAseq

RNA from three biological replicates of *B. pseudomallei *DD503 (a derivative of 1026b) grown in LB was extracted from cells in early logarithmic growth using RNAeasy (QIAgen, Valencia CA). Ribosomal RNAs were removed by 2 rounds of MicrobExpress (Ambion, Foster City CA). Each RNA preparation was used in individual cDNA synthesis reactions using SuperScript II (Invitrogen, Carlsbad CA) and sequenced individually in the Illumina Genome Analyzer (Illumina Technologies, San Diego CA) or SOLiD instruments with 100 or 50 bp reads, respectively. Data was analyzed using CLC Genomics Workbench allowing for 2 mismatches in each read and only one map location per read. Total gene expression was normalized according to the total number of reads in the library and the gene size, resulting in reads per kilobase per million reads (RPKM). Only genes that had more than 10 hits were considered to be expressed above the noise level.

## Results and Discussion

### Isolated and sequenced bacteriophages

**Figure 1:**
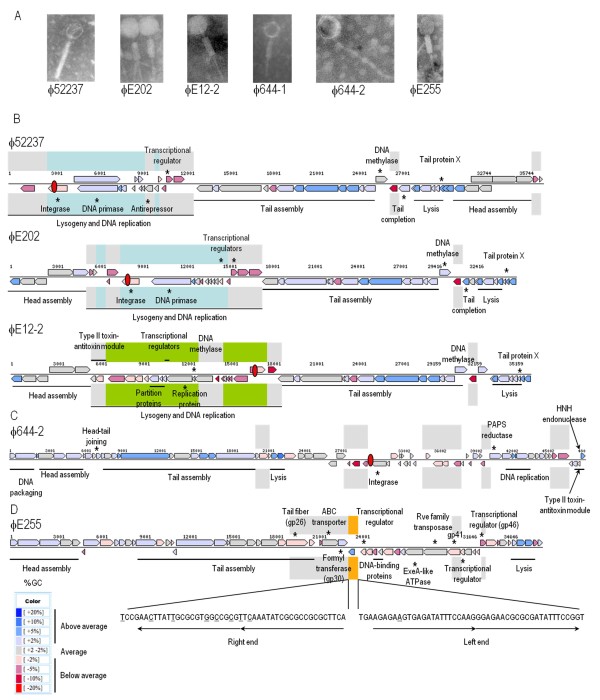
**Transmission electron micrographs (TEM) of the *Burkholderia *bacteriophages analyzed in this project and schematic illustrations of their genomes**. (A) TEM of bacteriophages negatively stained with 1% phosphotungstic acid. (B) Schematic illustrations of the P2-like Myoviridae genomes of ϕ52237, ϕE202, and ϕE12-2. Cyan shading represents sequences that are conserved in the subgroup A Myoviridae ϕ52237, ϕE202, and ϕK96243 and lime shading represents sequences that are conserved in the subgroup B Myoviridae ϕE12-2, GI15, and PI-E264-2. Gray shading represents sequences that are variably present in Myoviridae subgroups A and B. (C) Schematic illustration of the lambda-like Siphoviridae genome of ϕ644-2. Gray shading represents sequences that are unique to ϕ644-2. (D) Schematic illustration of the Mu-like Myoviridae genome of ϕE255. Gray shading represents sequences that are unique to ϕE255 and orange shading represents packaged host DNA. The 23-bp imperfect direct repeats at the left and right ends of the ϕE255 genome are shown and sequence differences with the repeat sequences of BcepMu are underlined. Genomic illustrations were obtained from the Integrated Microbial Genomes website http://img.jgi.doe.gov/cgi-bin/pub/main.cgi. Genes are shown as arrows that are pointing in their relative direction of transcription and are color coded based on their % GC composition (see scale at bottom). Individual genes with functional annotations are labeled and designated with an asterisk (*) while groups of genes with a common function are labeled and designated with a line. The locations of *att *sites are shown as red oblong circles. Nucleotide sequence numbering is shown above each genome.

Five bacteriophages were isolated from three *B. pseudomallei *and two *B. thailandensis *strains (Table 1A) when plaqued on *B. mallei *ATCC 23344 as a suitable host for bacteriophages [3,6,21]. Most *B. pseudomallei *and *B. thailandensis *strains only produced one phage, except for E12 and 644 which each produced at least two different phage particles. All of the bacteriophages contained long tails. Three were classified as P2-like viruses, one as a lambda-like virus, and one as a Mu-like virus. The bacteriophage genomes ranged in size from 35.7 to 48.7 Kb and contained from 47 to 71 genes. Specific details about each of these bacteriophages are provided below, representative images of each isolated bacteriphage are shown in Fig. 1A and other properties are described in Table 1A.

#### ϕ52237

*B. pseudomallei *Pasteur 52237 spontaneously produced a bacteriophage, designated ϕ52237 that formed uniform, slightly turbid plaques on *B. mallei *ATCC 23344, suggesting that this strain produces only one bacteriophage under the growth conditions used. While it is plausible that different bacteriophages might form plaques with the same morphology, here we assumed that similar plaques were formed by only one bacteriophage. Based on its morphotype, ϕ52237 can be classified as a member of the order Caudovirales and the family Myoviridae [38].

#### ϕE12-2

*B. pseudomallei *E12 spontaneously produced two bacteriophages, ϕE12-1 and ϕE12-2, that formed plaques on *B. mallei *ATCC 23344. ϕE12-1 produced turbid plaques of 0.5 to 1 mm in diameter and ϕE12-2 produced turbid plaques with a diameter of 1.5 to 2.0 mm. The purified plaques maintained their morphology following a further round of infection in the host suggesting that they were formed by two distinct bacteriophages. Approximately 10 pfu/ml of ϕE12-1 and ϕE12-2 were present in *B. pseudomallei *E12 culture supernatants. We were unable to isolate nucleic acid from ϕE12-1 and no further work was carried out on this bacteriophage. ϕE12-2 possessed an isometric head that was ~ 62 nm in diameter and a contractile tail that was ~ 152 nm long and ~ 21 nm in diameter (Fig. 1A). Similar to ϕ52237, ϕE12-2 can be classified as a member of the order Caudovirales and the family Myoviridae [38].

#### ϕ644-2

*B. pseudomallei *644 spontaneously produced 2 bacteriophages, ϕ644-1 and ϕ644-2, that formed plaques on *B. mallei *ATCC 23344. ϕ644-1 and ϕ644-2 produced plaques of different size and turbidity. ϕ644-2 was ten times more abundant in *B. pseudomallei *644 culture supernatants. Based on its morphology, ϕ644-2 can be classified as a member of the order Caudovirales and the family Siphoviridae [38]. The genome of ϕ644-1, a member of the Myoviridae family, could not be determined in this study.

#### ϕE255

*B. thailandensis *E255 spontaneously produced a bacteriophage, designated ϕE255, which formed turbid plaques with a diameter of ~ 0.5 mm on *B. mallei *ATCC 23344. No other plaque types were identified. Based on its morphotype, ϕE255 can be classified as a member of the order Caudovirales and the family Myoviridae [38].

#### ϕE202

*B. thailandensis *E202 spontaneously produced a bacteriophage, designated ϕE202, which formed turbid plaques on *B. mallei *ATCC 23344. No other plaque types were identified. ϕE202 production was increased 55-fold by brief exposure to UV light (data not shown). Based on its morphotype (Fig. 1A), ϕE202 can be classified as a member of the order Caudovirales and the family Myoviridae [38]. We examined the host range of ϕE202 using 17 *Burkholderia *species (Additional file 1, Table S1). Bacteriophage ϕE202 formed plaques on 9 of 10 natural *B. mallei *strains. It also formed plaques on a capsule-deficient mutant derived from ATCC 23344, DD3008 [39], suggesting that the capsular polysaccharide is not required for ϕE202 attachment. In contrast, two *B. mallei *strains that do not produce lipopolysaccharide (LPS) were resistant to plaque formation by ϕE202; NCTC 120 and DD110795 (a laboratory-passaged derivative of ATCC 15310), which suggests that LPS is a receptor, or co-receptor, for ϕE202. Given the >90% nucleotide identity of the tail assembly genes of the *Burkholderia *Myoviridae, it is likely that they all share the same receptor(s). Unlike other characterized *Burkholderia *Myoviridae (ϕE125, ϕ1026b), ϕE202 forms plaques on a species other than *B. mallei *(Additional file 1, Table S1), namely 3 strains of *B. pseudomallei*; NCTC 4845, STW 199-2, and STW 115-2. It is currently unknown why these *B. pseudomallei *strains exhibit plaque formation with ϕE202 while others do not. No other *Burkholderia *species examined formed plaques with ϕE202 (Additional file 1, Table S1).

### Genomic analysis of the Burkholderia phages

#### I. Myoviridae subgroup A and B

**Figure 2:**
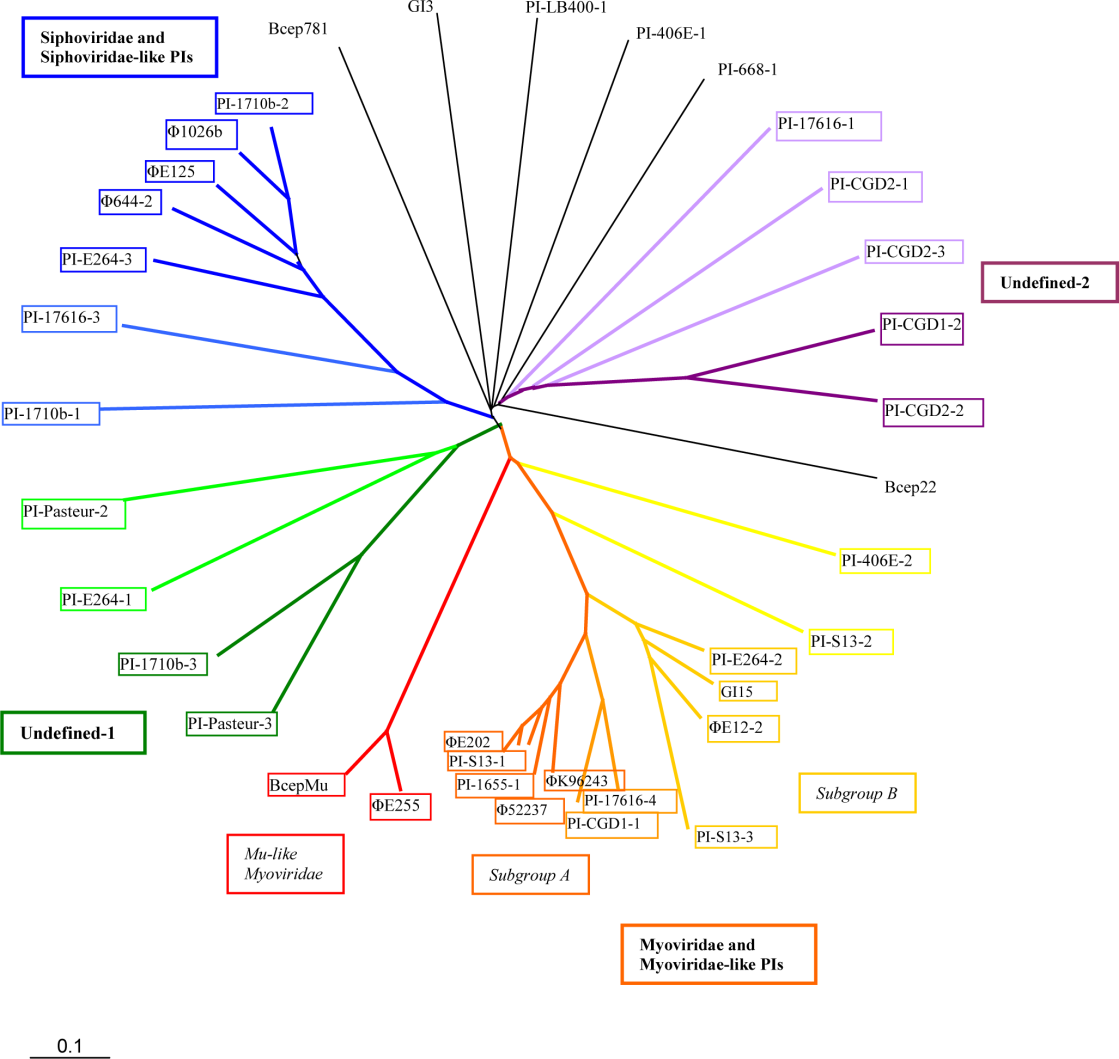
**Unrooted radial phylogenetic tree of the *Burkholderia *bacteriophages, putative prophages, and prophage-like regions analyzed in this study**. The tree was constructed from BLASTP distance matrix (cutoff E^-10^) using FITCH with the global and jumble options.

Based on sequence similarity, ϕ52237, ϕE202, and ϕK96243 belong to subgroup A of the Myoviridae and ϕE12-2, GI15, and PI-E264-2 to subgroup B (Fig. 2). Furthermore, the genomic structure of each of these are arranged in multigene "modules" that encode proteins involved in a common function, such as DNA packaging, head biosynthesis, tail biosynthesis, host lysis, lysogeny or DNA replication [40,41] (Fig. 1B). The relative order of these modules in ϕ52237, ϕE202, and ϕE12-2 is similar to that of bacteriophages P2 and ϕK96243 [42]. The order is also conserved in bioinformatically-identified prophage-like elements GI15 of *B. pseudomallei *K96243 and PI-E264-2 of *B. thailandensis *E264 (see below).

The modules for tail assembly, lysis, and head assembly of all Myoviridiae phages were highly conserved (Fig. 1B). However, the region encoding for lysogeny and DNA replication contained abundant genetic variability. Some of the genes within this region were present exclusively in either of the subgroups (Fig. 1B). This suggests that the mechanism of integration, regulation of excision, and/or replication of episomal bacteriophage DNA could be distinct for subgroup A and B Myoviridae. For example, subgroup A bacteriophage genomes encode DNA primase proteins which catalyze the synthesis of short RNA primers required for DNA replication by DNA polymerases (Fig. 1 B). Subgroup B bacteriophages, on the other hand, encode for ParA-like partitioning proteins which are ATPases involved in chromosome partitioning. In addition, subgroup B genomes encode replication gene A protein-like sequences. Members of this family of proteins are endonucleases which introduce single-strand nicks at or near the origin of replication (Fig. 1B).

Among the conserved regions, some segments are variably present in the bacteriophages and PIs (Fig. 1B). It is likely that these regions were acquired by recombination with unrelated bacteriophages (or prophages), and that these segments might be considered 'morons' [20]. This is supported by the fact that these regions exhibit a lower % GC content relative to the rest of the bacteriophage genomes (Fig. 1B), which suggests horizontal transfer of genetic information. Most of these novel genes encode conserved hypothetical proteins which have no defined functional activities, but share similarity with proteins in other bacteriophages. No obvious virulence factor genes are encoded by these bacteriophage genomes, which is consistent with a previous report on this topic [42]. Interestingly, ϕE12-2 gp6 and gp7 appear to encode a type II toxin-antitoxin (TA) addiction module (Fig. 1B - see below) [43]. Other novel proteins are encoded by the ϕ52237, ϕE202, and ϕE12-2 genomes (Fig. 1B), but no functions can be assigned to these gene products at this time.

The phage attachment sites (*attP*) of ϕ52237 and ϕE202 are found at the 3' ends of putative site-specific integrase genes (Fig. 1B) and are identical to each other. The nucleotide sequence of *attP *contained a 45-bp sequence that was identical to the 3' end of the phenylalanine tRNA (GAA) gene on chromosome 1 of *B. pseudomallei *K96243 (positions 145,379-145,454). This *attP *site is also utilized by ϕK96243 [3]. The integrase genes of these three subgroup A Myoviridae terminate with the tRNA (Phe) gene when integrated as prophages, but not when the bacteriophage genomes are episomal. Thus, following integration the integrase gene is partitioned into two fragments.

The ϕE12-2 *attP *site is located between gp24 and gp25 (5'-AATTTGACATAAGGTAAA-3') (Fig. 1B) and is identical to the sequence at both ends of GI15 in *B. pseudomallei *K96243 [3]. This integration site is present in an intergenic region on the *B. pseudomallei *genome and does not disrupt any obvious ORFs. This *attP *site does not have any homology to tRNAs. PI-E264-2 is also flanked by a similar sequence (5'-ATTTGACATAACGTAAA-3') in *B. thailandensis *E264, suggesting that it also uses this *attP*. No obvious integrase genes are encoded by ϕE12-2, GI15, or PI-E264-2, which suggests these subgroup B Myoviridae use a different mechanism of integration.

#### Mu-like phages

The ϕE255 genome shares ~ 90% nucleotide sequence identity with the genome of BcepMu, a Mu-like bacteriophage spontaneously produced by *Burkholderia cenocepacia *strain J2315 [29]. Similar to BcepMu, the ϕE255 genome can be divided into functional clusters from the left end to the right end of the linear phage genome: replication and regulation, host lysis, head assembly, and tail assembly (Fig. 1D). ϕE255 encodes a transposase with a Rve integrase domain (gp40, PFAM PF00665) that allows transposition as a mechanism of replication. Following replicative transposition, DNA is packaged into the bacteriophage heads using a *pac *site at the left end of the bacteriophage genome which allows 200-2,000 bp of flanking host DNA to also be packaged [29]. The genomic sequence of ϕE255 (accession number NC_009237) contains 467 bp of host DNA sequence (Bm ATCC23344). The left and right ends of the linear ϕE255 genome contain 23-bp imperfect direct repeats that could be recognized by gp40 during replicative transposition (Fig. 1D). These repeats are similar to those found at the ends of the BcepMu genome [29] and the nucleotide differences are underlined in Fig. 1D.

Three regions of the ϕE255 genome are not present in the BcepMu genome and appear to be ϕE255-specific (gray shading in Fig. 1D). The unique regions are found at the left and right ends of the ϕE255 genome, which is consistent with the location of unique sequences in BcepMu and other BcepMu-like prophages [29]. The two unique genes on the left side of the bacteriophage genome, gene*41 *and gene*46*, encode a conserved hypothetical protein and a lambda C1 repressor-like transcriptional regulator, respectively (Fig. 1D). These proteins are presumably involved in ϕE255 activation and/or replication. Five unique genes are encoded on the extreme right end of the ϕE255 genome, including genes *26*-*30 *(Fig. 1D). Gp26 encodes a putative tail fiber protein which presumably is required for attachment and probably provides host receptor specificity to this bacteriophage. It is interesting that this gene, and the downstream tail assembly chaperone protein (gp27), are the only tail assembly genes that are not conserved in BcepMu. This suggests that the BcepMu receptor(s) on *B. cenocepacia *is distinct from the ϕE255 receptor(s) on *B. thailandensis *and *B. mallei*. Furthermore, it suggests that the unique tail fiber protein and a tail assembly chaperone protein (gp27) were either acquired by ϕE255 via horizontal transfer or lost by BcepMu. Gp28 is a hypothetical protein with no functional prediction, but gp29 is a putative ABC (ATP-binding cassette) transporter protein (Fig. 1D). It is possible that ϕE255 gp29 is involved in the import of a nutrient or export of toxic metabolites that confers a selective advantage on the lysogen harboring it. On the other hand, *Lactococcus lactis *bacteriophage P335 [44] and *Streptococcus pyogenes *bacteriophage ϕNIH1.1 [45] also encode ABC transporters and these molecules may play an undefined role in the bacteriophage lifecycle. Finally, gp30 is a putative formyl transferase domain protein (Fig. 1D), a family of proteins involved in a variety of biochemical pathways, including *de novo *purine biosynthesis, methionyl-tRNA biosynthesis, and formate biosynthesis. None of these ϕE255 genes have homologs in any of the other phage/PI or *Burkholderia *genomes reported here or elsewhere.

#### Siphoviridae

The gene order and modular organization of the ϕ644-2 genome is reminiscent of lambdoid bacteriophages, including ϕ1026b and ϕE125 [6,21,46,47]. The ϕ644-2 genome harbors five regions that are specific to ϕ644-2 and contain a lower GC content than the rest of the ϕ644-2 genome, suggesting they may have been acquired horizontally from a novel source (gray shading in Fig. 1C). The thirteen novel genes present in these regions encode hypothetical proteins with no known function (gp22, gp23, gp24, gp33, gp34, gp35, gp46, gp47, gp48, gp49, gp55, gp66, and gp67). The genome also contains several interesting features, including a putative phosphoadenosine phosphosulphate (PAPS) reductase (gp56), a putative type II toxin-antitoxin module (gp69 and gp70), and a putative HNH endonuclease (gp71) that might be advantageous to the phage or its lysogen (Fig. 1C; discussed further below).

The ϕ644-2 genome contains ten base 3' single-stranded extensions on the left (3'-GCGGGCGAAG-5') and right (5'-CGCCCGCTTC-3') (Fig. 1C). In ϕE125, this sequence serves as a cohesive (*cos*) site [21], suggesting that ϕ644-2 uses the same *cos *site as ϕE125. The nucleotide sequence immediately downstream of gene*36*, which encodes a putative site-specific integrase, contained the candidate *attP *site of ϕ644-2. It is characterized by a 30-bp sequence that was identical to the 3' end of a 90-bp serine tRNA (GGA) gene on the *B. pseudomallei *K96243 small chromosome [3,4] (Fig. 1C). Interestingly, a 19-kb prophage-like island (GI13) is also integrated at this location in the *B. pseudomallei *K96243 genome [3,4], although there is no sequence similarity between the two elements.

### Inferred prophage islands

Twenty-four putative prophage or prophage-like regions were identified in 11 of the 20 *Burkholderia *strains (Table 1B). In addition, two GIs from K96243 (GI3 and GI15) were included in subsequent analysis since these also classify as putative prophage by our definition [3]. We call these regions prophage islands (PI) defined as regions of the genome that were found to contain most if not all of the elements characteristic of prophages (see Materials and Methods), but have not been isolated and experimentally characterized. Most *B. pseudomallei *and all *B. multivorans *strains were found to contain PIs; three were identified in *B. thailandensis *E264, one in *B. xenovorans *LB400, and none in any of the *B. mallei *strains. The three *B. thailandensis *E264 PIs, PI-E264-1, -2, and -3, correspond to *B. thailandensis *GI1, Bt GI13, and Bt GI12, respectively, as described by Yu et al [24], although the range of genes in the PIs described here differ slightly due to our criteria for inclusion. Similarly, PI-668-1 corresponds to GI15c from *B. pseudomallei *MSHR668 in Tuanyok et al [4]. As mentioned above, no PIs were detected in *B. pseudomallei *1106a/b, although phage-like remnants were found in these strains. Overall, seventeen of the 24 identified regions were located on chromosome I of the respective bacterial strain, and all but five were putative prophages (i.e., most likely to be active prophages containing all of the prophage-like elements described in the Materials and Methods). Of the seven regions located on chromosome II, one (PI-E264-3) was classified as a putative prophage, while the remaining six were designated prophage-like.

### Paired strains B. pseudomallei 1710a/b and B. pseudomallei 1106a/b

The two pairs *B. pseudomallei *1710a/b and *B. pseudomallei *1106a/b represent two bacterial strains isolated at different time points from the same two infected patients, isolated from the primary infection (a) and the relapse (b). We hypothesized that difference/s in sequence relating to the relapse or host selection would be detected, either in the form of SNPs/indels or as variation in the phage harbored within each strain. Three PIs were identified in each of the *B. pseudomallei *1710 strains. PI-1710a/b-1 is immediately followed by PI-1710a/b-2 on chromosome I, separated by a tRNA pseudogene in each strain. This region is described as GI6b in Tuanyok et al. [4]. PI-1710a/b-3 is located further downstream on chromosome I. All three regions are nearly identical, averaging 98.4, 97.7, and 96.6% identity over 98.2, 97.1, and 96.2% of length (for -1, -2, and -3, respectively). PI-1710a-1 and PI-1710b-1 are 41.3 and 41.4 kb in length, respectively, and both are bounded by tRNA-Pseudo-2 and a 23 bp exact repeat of the 3' end of this tRNA. Both PI-1710a-2 and PI-1710b-2 are 60.6 kb in size and are bounded by tRNA-Pro-2 and a 49 bp exact repeat. The prophage-like regions in both strains (PI-1710a-3, PI-1710b-3) are defined by the presence of a phage integrase at the 3' end by tRNA-Thr-3, with several viral-like proteins immediately upstream, but no repeat region could be identified to define the 5' end. Both are 62.8 kb. Since the three prophage islands are nearly identical between *B. pseudomallei *1710a and *B. pseudomallei *1710b, from here on we will only refer to *B. pseudomallei *1710b and associated prophage islands. These results indicate that the prophage in Bp 1710a/b were not excised and did not experience any significant changes even after passage through a host.

By the definitions set forth for prophage islands given in this work, no PIs were identified in either of the *B. pseudomallei *1106 strains. Tuanyok et al. [4] described 16 genomic islands in 1106a, one of which was identified as a putative prophage (GI10.2; BURPS1106A_3666 - 3701). However, this region also contains three transposases, and so was not considered in the analysis reported here.

### Bacteriophage clusters

Results from the Dotter analysis allowed a preliminary clustering of prophages and prophage-like regions. These groups were further refined by examination of BLASTP protein distance data, resulting in the clustering of 32 of the 37 PIs and prophages into each of four groups (data not shown). Cluster composition was very similar between the three BLASTP-distance FITCH trees and agreed with DOTTER results, although branch positions varied slightly (Fig. 2). Seven prophages/PIs clustered into the Siphoviridae-like group, so named because of the inclusion of the previously published bacteriophages ϕ1026b [6] and ϕE125 [21]. Bacteriophage ϕ644-2, described in this study, is also a member of this group (Fig. 2). Prophages in this group have long non-contractile tails and termini with cohesive ends. The *cos *site, present in ϕ1026b and ϕE125, was identified in all other members of this group.

The Myoviridae-like group consists of 15 prophages/PIs (Fig. 2). Phages in this group, identified by the inclusion of ϕK96243 (GI2) [3] and ϕ52237, typically have contractile tails and terminal repeats [48]. Three subgroups were identified within the Myoviridae-like class (Fig. 2). Subgroup A contains ϕK96243, ϕ52237, ϕE202, and four other prophages/PIs. Bacteriophage ϕE12-2 and five prophages/PIs clustered to form subgroup B, including two (PI-406E-2 and PI-S13-2) which appear to be more distantly related. The Mu-like Myoviridae group contains only two prophages: BcepMu [29] and ϕE255. Both left and right phage ends at the host/phage junction in BcepMu [29] were located at the ends of ϕE255, with 95% and 91% identity, respectively. No significant identity was found between either of the two Mu-like prophages and any of the other prophages or prophage-like sequences.

Two undefined groups were also identified: undefined-1 contains four PIs, and undefined-2 has five (Fig.2). Interestingly, undefined-2 contains five of the eight PIs identified in the three *B. multivorans *strains. Finally, six sequences had no significant similarity to any other sequence and were thus considered unclustered, including PI-668-1, PI-406E-1, PI-LB400-1, GI3, Bcep22 and Bcep781.

### Burkholderia bacteriophages are populated by morons

**Figure 3:**
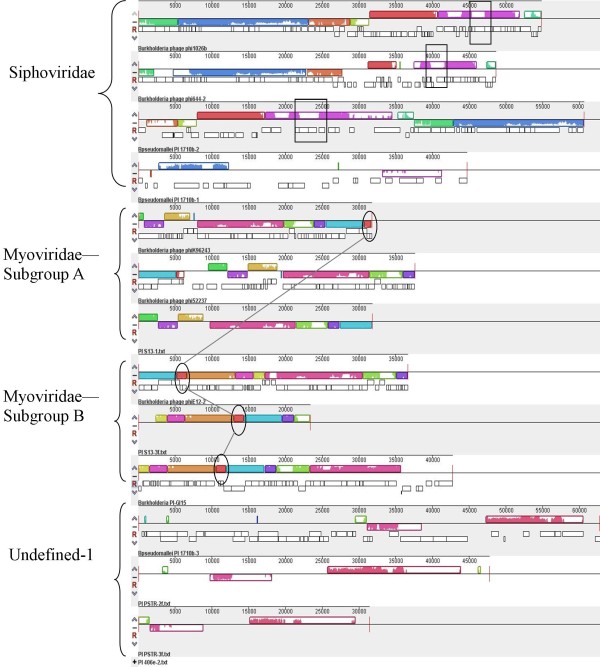
**Comparative alignment of *B. pseudomallei *phage and prophage-like regions**. Colors indicate local collinear blocks (LCB or synteny blocks) between phages and PI, the level of similarity between sequences is directly proportional to the height of the colored bars within the LCB. Rectangles highlight hotspots where different morons integrated in each of the genomes. Ovals highlight the same moron which integrates at the same location in each genome, but is differentially present among genomes within the same groups and is present in different phylogenetic groups. Genome comparisons and LCB calculations were performed using Mauve software [34].

Genomic comparisons of all the phages in each class revealed that the genomes are arranged in mosaic structures. Each of the phylogenetic classes of phages contains distinct local collinear blocks (LCB), also called synteny blocks, which are differentially present among the phages in that group (Fig. 3). Within each group, the synteny blocks are shuffled among the genomes (Fig. 3), suggesting that several of the phages have undergone dramatic genomic rearrangements. In several instances, synteny blocks were either abruptly cut off at one end, or a predicted ORF appeared within the block in one genome, but was absent in other phages (see white areas within LCB in Fig. 3). We hypothesized that these randomly occurring ORFs could be morons, i.e. genetic elements that integrate between adjacent phage genes, which may confer some additional fitness to the bacteriophage [20]. Aside from being inserted among phage genes, morons usually (i) have their own transcriptional control system (a promoter, operator, and/or terminator), (ii) have a different GC content than the rest of the phage, and (iii) may be found in more than one class of phage [20].

**Figure 4:**
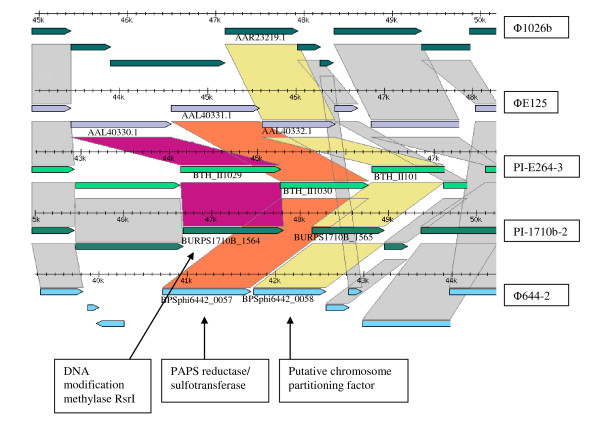
**Regional sequence alignments of Siphoviridae-like prophages**. Comparative genomic analysis of siphoviridae-like prophages and PIs detailing morons encoding DNA methylase RsrI, PAPS reductase/sulfotransferase, and putative chromosome partitioning factor. Gray shading represents conservation at greater than 90% identity among all genomes. Mauve or orange shading represents conservation at 90% identity in a subset of genomes.

In most cases, the randomly occurring ORFs detected by LCB analysis of the *Burkholderia *phages displayed at least 2 of the characteristics of morons (data not shown), and thus we classified them as morons themselves. In total, 17 different morons were identified among the phages and prophages-like islands (Table 2). Some of the PI/phages had as many as 12 morons, while some had as few as 2 (Table 2). Among different phage genomes, morons appeared adjacent to homologous genes across phylogenetic groups (see ovals in Fig. 3), and in many cases, some regions seemed to be hotspots for moron entry since different morons were detected at the same location (see below; Fig. 4).

**Table 2:** Morons identified among phages and PI in *Burkholderia *species.

Predicted Moron Function*	Species with closest BLAST hit	Phages													
Abortive infection resistance	*Thiobacillus denitrificans*	PI-1710b-3													
Addiction module (killer - antidote)	*Rhodopseudomo-nas palustris*	ϕK96243	ϕE12-2	GI15	PI-S13-3										
Aromatic compound degradation (2 genes)	*Sphingomonas wittichii*	GI3													
Colicin (cvaB)	*Psedomonas aeruginosa*	PI-Pasteur-3													
Colicin (ompA)	*Cupriavidus taiwanensis*	PI-1710b-3	PI-Pasteur-3												
CW surface anchor 1	*Cupriavidus metallidurans*	PI-Pasteur-2													
CW surface anchor 2 proteophosphoglycan 5	*Cupriavidus metallidurans*	PI-1710b-3	PI-Pasteur-2	PI-Pasteur-3											
HNH motif (DNA endonuclease)	*Xanthomonas albilineans*	ϕ644-2	ϕ1026b	ϕE125	PI-1710b-2										
ImpA family protein	*Ralstonia solanacearum*	PI-1710b-3	PI-Pasteur-3												
PAPS reductase	*Verminephrobacter eiseniae*	ϕE125	ϕ644-2	PI-E264-3											
Patatin-like phospholipase (1)	*Frankia sp*.	PI-1710b-2													
Patatin-like phospholipase (2)	*Alteromonas macleodii*	PI-LB400-1													
Phage growth limitation system (pglY, pglZ)	*Polaromonas naphthalenivorans*	PI-E264-1													
Pyocin repressor protein (PrtR)	*Ralstonia picketti*	PI-CGD1-2	PI-17616-1												
Pyocin-related (R2_PP-tail formation)(1)	*Xanthomonas oryzae*	ϕK96243	PI-17616-4	PI-1655-1	ϕE202	ϕ52237	PI-CGD1-1	PI-264-4	ϕE12-2	GI15	PI-S13-1	PI-S13-3	PI-406E-2	ϕE265	BcepMu
Pyocin-related (R2_PP-tail formation)(2)	*Azotobacter vinelandii Phage*	ϕK96243	PI-17616-4	PI-1655-1	ϕE202	ϕ52237	PI-CGD1-1	PI-S13-1	ϕE12-2	GI15	PI-E264-2	PI-S13-3	PI-406E-2		
Pyocin-related (TraC domain)	*Pseudomonas fluorescens*	PI-406E-2													
Reverse transcriptase (UG1)	*Ralstonia eutropha*	GI3													
Reverse transcriptase (UG3 & 8)	*Providencia stuartii*	GI3													
Soluble lytic murein trans glycolase	*Sideroxydans lithotrophicus*	ϕE255	BcepMu												
TA system (relE)	*Beggiatoa sp. PS*	ϕ1026b	ϕE125	ϕ644-2	PI-1710b-2										
TI secretion (tolC)	*Psedomonas aeruginosa*	PI-Pasteur-3													
TII secretion (eha)	*Chromobacterium violaceum*	ϕE255	BcepMu												
TIII restriction-modification system (2 genes)	*Aromatoleum aromaticum*	PI-1710b-3													
Type I restriction-modification system (4 genes)	*Acidovorax sp*.	PI-Pasteur-3													

*Morons were identified as described in Methods. Phages listed in each column contain the predicted moron function. Non-*Burkholderia *species that have the closest protein as identified by BLASTp (E value less than 10^-3^) are presented.

Analysis of predicted functions of the *Burkholderia *morons shows that several of these proteins may enhance bacteriophage fitness, and thus replication, as proposed for other morons [20]. For example, two different morons containing toxin-antitoxin modules were found among the Myoviridae and Siphoviridae groups (Table 2). Interestingly, the T-A module in the Myoviridae phages is similar to two modules present in other *B. pseudomallei *and even *B. mallei *strains in regions containing phage remnants (data not shown), suggesting that this moron can persist even after the phage has been excised from the genome.

Several of the morons appeared to enhance the metabolic versatility of the host: aromatic compound degradation, iron transport and acquisition, and sulfate assimilation (Table 2). For example, it has been suggested that the PAPS reductase gene, which functions in the assimilatory sulfate reduction pathway, could serve as a fitness factor under conditions of iron limitation for the lysogens that harbor prophages encoding this enzyme [42]. PAPS reductase genes were identified in three members of the Siphoviridae-like group, ϕE125, ϕ644-2 and PI-E264-3 (Fig. 4), and in the Myoviridae-like B subgroup member PI-E264-2. The PAPS reductase moron incorporated between two highly conserved phage genes (Fig. 4) at a location that appears to be an insertion hotspot, since the other members of this group contain different morons (Fig. 4 and rectangles in Fig. 3).

Other morons appear to be associated with enhanced host or bacteriophage competitiveness. For example, morons within the Myoviridae, Undefined-1, Undefined-2, and Siphoviridae encode for the production of toxins that inhibit the growth of competing bacterial strains (bacteriocins) and/or their associated translocation mechanisms (Table 2). Other morons could prevent infection of their host by other phage, these include morons that encode for site-specific endonucleases, DNA methylases, restriction-modification systems, phage abortive infection resistance, and phage-growth limiting genes. Although we could not confirm that GI3 from K96243 contains morons (since LCB analysis was limited to those PIs that formed clusters), two separate reverse-transcriptase (RT) modules are encoded in this PI. Many phage-encoded RT described to date also function in phage resistance by directly targeting other phage DNA.

Lastly, some of the morons encode for proteins associated with bacterial virulence (Table 2). Two different morons encode patatin-like phospholipases (PTP), which in *P. aeruginosa *can act as cytotoxins necessary for virulence in amoeba and contribute to lung injury in a mouse model [18,49,50]. Moreover, a prophage-encoded phosholipase in group A *Streptococcus *also appears to enhance virulence and its expression results in more severe disease [49]. Two other morons encode for a proteophosphoglycan and a lytic transglycosylase, both of which have been associated with virulence in other pathogens [51]. Thus, some phages in *Burkholderia *spp. might also be implicated in enhanced virulence.

### Moron and phage genes are differentially expressed in Bp DD503

We performed transcription analysis using RNAseq to determine to what extent phage genes and morons are expressed in ϕ1026b. The results demonstrate that most phage genes are normally not expressed in rich laboratory growth conditions (Table 3), and allowed us to determine at least one putative repressor that maintains such regulation. For ϕ1026b, the candidate repressor gene (*phi1026bp79*) had a very high expression value which was 4-times higher than any of the phage structural or replication genes, (Table 3). The protein (gp78) contains a divergent AAA domain involved in ATP-binding and has similarities to other proteins annotated as transcriptional factors, such as YP_002500546 from *Methylobacterium nodulans *ORS 2060. Most of the phage morphogenesis and replication genes are only expressed at low levels, with many genes (54 of 89 genes) not having any detectable expression (Table 3). In many phages, gene expression and lysogenic conversion occur only when the levels of the repressor protein drop below a certain threshold. None of the other phages identified in this study had proteins with homology to this putative repressor suggesting that their mechanisms of regulation are different.

**Table 3:** RNASeq analysis of gene expression of phage genes in Bp DD503.

Gene	Annotation	Expression value (RPKM)*
phi1026bp03	putative portal protein	3,601
phi1026bp05	putative major capsid protein	4,743
phi1026bp14	putative tail length tape measure protein	1,038
phi1026bp16	hypothetical protein	3,986
**phi1026bp27**	**putative DNA adenine methylase**	**21,563**
**phi1026bp28**	**hypothetical protein**	**199,000**
**phi1026bp29**	**PAAR repeat-containing protein**	**186,000**
**phi1026bp30**	**VRR-NUC domain protein**	**132,500**
phi1026bp31	hypothetical protein	77,624
phi1026bp32	hypothetical protein	8,751
phi1026bp33	hypothetical protein	17,084
phi1026bp34	putative site-specific integrase	5,746
phi1026bp36	hypothetical protein	23,220
phi1026bp37	hypothetical protein	80,994
phi1026bp38	hypothetical protein	16,224
phi1026bp44	hypothetical protein	2,494
phi1026bp48	hypothetical protein	2,501
phi1026bp51	hypothetical protein	26,846
phi1026bp59	putative LysR family transcriptional regulator	18,809
phi1026bp60	putative major facilitator family permease	29,669
phi1026bp61	hypothetical protein	33,472
phi1026bp62	hypothetical protein	46,783
phi1026bp63	hypothetical protein	10,273
phi1026bp64	hypothetical protein	219,500
phi1026bp65	hypothetical protein	220,000
phi1026bp78	hypothetical protein	4,184
phi1026bp79**	*putative transcriptional regulator*	*59,976*
**phi1026bp81**	**XRE familiy putative transcriptional regulator**	**53,561**
**phi1026bp82**	**addiction module toxin, RelE/StbE family**	**92,307**

*Genes in **bold **belong to morons. Only genes with 10 or more reads are displayed, genes with fewer than 10 reads are considered non-expressed since they are not above noise level. Expression values are measured as reads per kilobase of coding sequence per million reads (RPKM). Number of reads and expression values are from one Illumina run, but are representative of 3 runs.

**Candidate phage repressor.

In addition to the highly expressed repressor, several of the morons in ϕ1026b were also expressed, consistent with the notion that morons are differentially regulated from the rest of the prophage genes as proposed by Hendrix *et al *[20]. The toxin-antidote morons were highly expressed, with the toxin gene (*phi1026bp82*) 1.5-fold higher than the antidote gene (*phi1026bp81*; Table 3). The DNA methylase and restriction modification moron (*phi1026bp28-30*), major facilitator permease, LysR transcriptional regulator and other morons with hypothetical proteins present among the genome were also highly expressed (Table 3) independently of the phage genes in their vicinity, further suggesting that phages represent a rich source of fitness factors that benefit the host even while the phage genes are repressed.

## Conclusions

The vast diversity in pathogenicity, clinical presentation, and living environments that exists within and between the *Burkholderiae *can be attributed at least in part to the presence of prophages and prophage-like elements within the genomes of these microbes. In this report we have characterized and classified 37 prophages, putative prophages, and prophage-like elements identified from several *Burkholderia *species and strains within species. Five spontaneously produced bacteriophages of lysogenic *B. pseudomallei *and *B. thailandensis *were isolated and characterized, including their host range, genome structure, and gene content. Using bioinformatic techniques, 24 putative prophages and prophage-like elements were identified within whole genome sequences of various *Burkholderia *species. Interestingly, while putative prophages were found in all but one of the *B. pseudomallei *strains none were detected in any of the *B. mallei *strains searched. The *B. mallei *genome is nearly identical to that of *B. pseudomallei*, differing by several contiguous gene clusters in *B. pseudomallei *that appear to have been deleted from *B. mallei*, and it is hypothesized that *B. mallei *evolved from a single *B. pseudomallei *strain [8,9]. If true, it is likely that this *B. pseudomallei *strain had at least one prophage within its genome that was excised from *B. mallei *leaving behind a toxin-antitoxin module. The prophage excision was part of a major host adaptation in *B. mallei *that also removed ~1200 other genes [8]. In addition, *B. mallei *is largely confined to a mammalian host in nature and is less likely to be exposed to new bacteriophages in this niche relative to other *Burkholderia *species that are commonly found in the soil/plant rhizosphere. Taken together, prophage elimination and limited prophage acquisition probably account for the lack of functional prophages in the *B. mallei *genome.

Sequences of the five isolated and sequenced bacteriophages, the 24 inferred prophages, and eight previously published *Burkholderia *prophages or putative prophages were classified based on nucleotide and protein sequence similarity, and an unrooted radial tree was constructed to estimate genetic relatedness between them. Several sequences could be classified as Siphoviridae-like, Myoviridae-like, or Mu-like Myoviridae based on similarity to phages known to be members of these groups. Additionally, two novel groups were detected, and five prophages/PIs could not be grouped with other phages. For the most part the phage groups were represented across all species and strains, with the notable exception of the undefined-2 group, which is composed primarily of *B. multivorans*-derived PIs (five from *B. multivorans*, one from *B. pseudomallei*), albeit loosely related. Further work that includes prophages derived from environmental and clinical isolates from other *Burkholderia *species as well as from other microbes is needed to refine these relationships.

*Burkholderia *spp. are responsible for a number of potentially devastating infectious diseases for which no vaccines currently exist. The presence of a wide variety of bacteriophages within these bacteria opens the possibility that phage therapy may be developed to augment present antibiotic treatments. We present here a detailed comparative analysis of gene content within and between groups of bacteriophages, putative prophages, and prophage-like regions in various *Burkholderia *species and strains. Several interesting genes and gene groups associated with pathogenicity and various metabolic functions were identified within specific groups. This study provides the first estimate of the relative contribution of prophages to the vast phenotypic diversity found among the *Burkholderiae*.

## Authors' contributions

CMR and LL conducted data analyses, comparative genomics, and wrote manuscript. LB and JI participated in bioinformatic and genomic analysis. RU and DD isolated and characterize phages and isolated phage DNA. MS isolated RNA for transcritpome analysis. WCN and DD conceived of the study, participated in its design and coordination, and helped draft manuscript. All authors have read and approved the final manuscript.

## Supplementary Material

Additional file 1 **Additional tables**. This file contains Tables S1 and S2 that describe the host range of phiE202 and all the strains that were used to search for prophages. Table S1. Bacterial strains used to examine the host range of bacteriophage phiE202. Table S2. Burkholderia strains searched for putative prophage. Click here for file
