# Supplementary material for: Genetic and phenotypic diversity in Burkholderia: contributions by prophage and phage-like elements
Source: BMC Microbiol. 2010 Jul 28;10:202. doi: 10.1186/1471-2180-10-202 (PMC2920897; doi:10.1186/1471-2180-10-202)
Supplement: Additional file 1 — Additional tables. This file contains Tables S1 and S2 that describe the host range of phiE202 and all the strains that were used to search for prophages. Table S1. Bacterial strains used to examine the host range of bacteriophage phiE202. Table S2. Burkholderia strains searched for putative prophage. [file 1471-2180-10-202-S1.PDF]

**Additional file**

Title: Additional tables.
Description: This file contains Tables S1 and S2 that describe the host range of phiE202 and all the strains that were used to search for prophages.

**Table S1. Bacterial strains used to examine the host range of bacteriophage ϕE202.**

| Bacterial strain | Source or reference | Plaque formation |
|---|---|---|
| *Burkholderia mallei* | | |
| NCTC 120 | NCTC | - |
| NCTC 10248 | " | + |
| NCTC 10229 | " | + |
| NCTC 10260 | " | + |
| NCTC 10247 | " | + |
| NCTC 3708 | " | + |
| NCTC 3709 | " | + |
| ATCC 23344 | ATCC | + |
| ATCC 10399 | " | + |
| ATCC 15310 | " | + |
| DB110795 | [1] | - |
| DD3008 | [2] | + |
| *Burkholderia pseudomallei* | | |
| 316c | [3] | - |
| NCTC 4845 | NCTC | + |
| 1026b | [4] | - |
| WRAIR 1188, USAMRU Malaysia 32, Pasteur 52237, STW 176, STW 152, STW 102-3, STW 35-1 | [5] | - |
| STW 199-2 | [5] | + |
| STW 115-2 | [5] | + |
| E203 | [6, 7] | - |
| *Burkholderia thailandensis* | | |
| E125, E202, E275, E251, E264 | [6, 7] | - |
| *Burkholderia cepacia* (genomovar I) | | |
| LMG 1222 | [8] | - |
| *Burkholderia multivorans* | | |
| C5568 | P. Sokol, U of Calgary | - |
| LMG 18823 | [8] | - |
| *Burkholderia cepacia* (genomovar III) | | |
| LMG 18863 | [8] | - |
| 715j | [9] | - |
| *Burkholderia stabilis* | | |
| LMG 07000 | Pamela A. Sokol, University of Calgary | - |

| | | |
|---|---|---|
| *Burkholderia vietnamiensis* | | |
| LMG 16232, LMG 10929 | [8] | - |
| *Burkholderia gladioli* | | |
| 2-72, 2-75, 4-54, 5-62 | [10] | - |
| *Burkholderia uboniae* | | |
| EY 3383 | [11] | - |
| *Burkholderia cocovenans* | | |
| ATCC 33664 | ATCC | - |
| *Burkholderia pyrrocinia* | | |
| ATCC 15958 | ATCC | - |
| *Burkholderia glathei* | | |
| ATCC 29195 | ATCC | - |
| *Burkholderia caryophylli* | | |
| Pc 102 | N.W. Schaad, USDA | - |
| *Burkholderia andropogonis* | | |
| PA-133 | N.W. Schaad, USDA | - |
| *Burkholderia kururiensis* | | |
| KP23 | [12] | - |
| *Burkholderia* spp. | | |
| 2.2N | [13] | - |
| T-22-8A | J.O. Falkinham III, Virginia Tech | - |

**Table S2. *Burkholderia* strains searched for putative prophage.**

| Strain | Accession # | Source | Description |
|---|---|---|---|
| *B. pseudomallei:* | | | |
| 1655 | NZ_AAHR00000000 | Australia | Chronic pulmonary melioidosis (sputum isolate) |
| 1710a | NZ_AAHS00000000 | Thailand | Primary melioidosis (blood culture) |
| 1710b | NC_007434, NC_007435 | Thailand | First relapse melioidosis, fatal (blood culture) |
| 668 | NC_009074, NC_009075 | Australia | Severe melioidosis encephalomyelitis (blood culture) |
| 1106a | NC_009076, NC_009078 | Thailand | Primary melioidosis (liver abscess) |
| 1106b | NZ_AAMB00000000 | Thailand | First relapse melioidosis, nonfatal (liver abscess) |
| Pasteur 6068 | NZ_AAHV00000000 | Viet Nam | 1950's isolate; used in pathogenesis studies |
| 406E | NZ_AAMM00000000 | Thailand | Disseminated melioidosis, fatal (toe swab) |
| S13 | NZ_AAHW00000000 | Singapore | Unique capsule; mucoid environmental strain |
| *B. mallei* | | | |
| ATCC 23344 | NC_006348, NC_006349 | Burma | Post-mortem culture [14] |
| ATCC 10399 | NZ_AAHN00000000 | China | Virulent |
| NCTC 10229 | NC_008836, NC_008835 | Hungary | Virulent |
| NCTC 10247 | NC_009080, NC_009079 | Turkey | Unknown passage history; avirulent in hamsters |
| 2002721280 | NZ_AANX00000000 | Pasteur Institute | avirulent in hamster model |
| SAVP1 | NC_008785, NC_008784 | ? | |
| *B. multivorans* | | | |
| ATCC 17616 | NC_010084, NC_010086, NC_010087 | | Associated with infections in cystic fibrosis patients |
| CGD1 | NZ_ACFB00000000 | | Associated with infections in cystic fibrosis patients |
| CGD2 | NZ_ACFC00000000 | | Associated with infections in cystic fibrosis patients |
| *B. thailandensis* | | | |
| E264 | NC_007651, NC_007650 | Thailand | Environmental isolate [15] |
| *B. xenovorans* | | | |
| LB400 | NC_007951, NC_007952, NC_007952 | New York, USA | PCB-contaminated landfill [16] |

## REFERENCES


1. Woods DE: **The use of animal infection models to study the pathogenesis of melioidosis and glanders**. *Trends Microbiol* 2002, **10**(11):483-484; discussion 484-485.
2. DeShazer D, Waag DM, Fritz DL, Woods DE: **Identification of a Burkholderia mallei polysaccharide gene cluster by subtractive hybridization and demonstration that the encoded capsule is an essential virulence determinant**. *Microb Pathog* 2001, **30**(5):253-269.
3. Godfrey AJ, Wong S, Dance DA, Chaowagul W, Bryan LE: **Pseudomonas pseudomallei resistance to beta-lactam antibiotics due to alterations in the chromosomally encoded beta-lactamase**. *Antimicrob Agents Chemother* 1991, **35**(8):1635-1640.
4. DeShazer D, Brett PJ, Carlyon R, Woods DE: **Mutagenesis of Burkholderia pseudomallei with Tn5-OT182: isolation of motility mutants and molecular characterization of the flagellin structural gene**. *J Bacteriol* 1997, **179**(7):2116-2125.
5. Finkelstein RA, Atthasampunna P, Chulasamaya M: **Pseudomonas (Burkholderia) pseudomallei in Thailand, 1964-1967: geographic distribution of the organism, attempts to identify cases of active infection, and presence of antibody in representative sera**. *Am J Trop Med Hyg* 2000, **62**(2):232-239.
6. Smith MD, Wuthiekanun V, Walsh AL, White NJ: **Quantitative recovery of Burkholderia pseudomallei from soil in Thailand**. *Trans R Soc Trop Med Hyg* 1995, **89**(5):488-490.
7. Wuthiekanun V, Smith MD, Dance DA, White NJ: **Isolation of Pseudomonas pseudomallei from soil in north-eastern Thailand**. *Trans R Soc Trop Med Hyg* 1995, **89**(1):41-43.
8. Mahenthiralingam E, Coenye T, Chung JW, Speert DP, Govan JR, Taylor P, Vandamme P: **Diagnostically and experimentally useful panel of strains from the Burkholderia cepacia complex**. *J Clin Microbiol* 2000, **38**(2):910-913.
9. McKevitt AI, Bajaksouzian S, Klinger JD, Woods DE: **Purification and characterization of an extracellular protease from Pseudomonas cepacia**. *Infect Immun* 1989, **57**(3):771-778.
10. Segonds C, Heulin T, Marty N, Chabanon G: **Differentiation of Burkholderia species by PCR-restriction fragment length polymorphism analysis of the 16S rRNA gene and application to cystic fibrosis isolates**. *J Clin Microbiol* 1999, **37**(7):2201-2208.
11. Yabuuchi E, Kawamura Y, Ezaki T, Ikedo M, Dejsirilert S, Fujiwara N, Naka T, Kobayashi K: **Burkholderia uboniae sp. nov., L-arabinose-assimilating but different from Burkholderia thailandensis and Burkholderia vietnamiensis**. *Microbiol Immunol* 2000, **44**(4):307-317.
12. Zhang H, Hanada S, Shigematsu T, Shibuya K, Kamagata Y, Kanagawa T, Kurane R: **Burkholderia kururiensis sp. nov., a trichloroethylene (TCE)-degrading bacterium isolated from an aquifer polluted with TCE**. *Int J Syst Evol Microbiol* 2000, **50 Pt 2**:743-749.
13. Cain CC, Henry AT, Waldo RH, 3rd, Casida LJ, Jr., Falkinham JO, 3rd: **Identification and characteristics of a novel Burkholderia strain with broad-spectrum antimicrobial activity**. *Appl Environ Microbiol* 2000, **66**(9):4139-4141.

14. Nierman WC, DeShazer D, Kim HS, Tettelin H, Nelson KE, Feldblyum T, Ulrich RL, Ronning CM, Brinkac LM, Daugherty SC *et al*: **Structural flexibility in the Burkholderia mallei genome**. *Proc Natl Acad Sci U S A* 2004, **101**(39):14246-14251.
15. Kim HS, Schell MA, Yu Y, Ulrich RL, Sarria SH, Nierman WC, DeShazer D: **Bacterial genome adaptation to niches: divergence of the potential virulence genes in three Burkholderia species of different survival strategies**. *BMC Genomics* 2005, **6**:174.
16. Chain PS, Denef VJ, Konstantinidis KT, Vergez LM, Agullo L, Reyes VL, Hauser L, Cordova M, Gomez L, Gonzalez M *et al*: **Burkholderia xenovorans LB400 harbors a multi-replicon, 9.73-Mbp genome shaped for versatility**. *Proc Natl Acad Sci U S A* 2006, **103**(42):15280-15287.
